# Human Milk Oligosaccharide Concentrations and Infant Intakes Are Associated with Maternal Overweight and Obesity and Predict Infant Growth

**DOI:** 10.3390/nu13020446

**Published:** 2021-01-29

**Authors:** Jessica L. Saben, Clark R. Sims, Ann Abraham, Lars Bode, Aline Andres

**Affiliations:** 1 J.L.S. Scientific Consulting, L.L.C., Thornton, CO 80229, USA; jlsaben@gmail.com; 2 Arkansas Children’s Nutrition Center, Little Rock, AR 72202, USA; crsims@uams.edu; 3 Department of Pediatrics, University of Arkansas for Medical Sciences, Little Rock, AR 72205, USA; 4 Larsson-Rosenquist Foundation Mother-Milk-Infant Center of Research Excellence (MOMI CORE), Department of Pediatrics, University of California San Diego, La Jolla, CA 92093, USA; annmary_101@yahoo.com (A.A.); lbode@health.ucsd.edu (L.B.)

**Keywords:** lactation, HMO, maternal obesity, breastfeeding, breastmilk, DOHaD, developmental programing, adiposity, infant

## Abstract

Human milk oligosaccharides (HMOs) are bioactive molecules playing a critical role in infant health. We aimed to quantify the composition of HMOs of women with normal weight (18.5–24.9 kg/m^2^), overweight (25.0–29.9 kg/m^2^), or obesity (30.0–60.0 kg/m^2^) and determine the effect of HMO intake on infant growth. Human milk (HM) samples collected at 2 months (2 M; *n* = 194) postpartum were analyzed for HMO concentrations via high-performance liquid chromatography. Infant HM intake, anthropometrics and body composition were assessed at 2 M and 6 M postpartum. Linear regressions and linear mixed-effects models were conducted examining the relationships between maternal BMI and HMO composition and HMO intake and infant growth over the first 6 M, respectively. Maternal obesity was associated with lower concentrations of several fucosylated and sialylated HMOs and infants born to women with obesity had lower intakes of these HMOs. Maternal BMI was positively associated with lacto-N-neotetraose, 3-fucosyllactose, 3-sialyllactose and 6-sialyllactose and negatively associated with disialyllacto-N-tetraose, disialyllacto-N-hexaose, fucodisialyllacto-N-hexaose and total acidic HMOs concentrations at 2 M. Infant intakes of 3-fucosyllactose, 3-sialyllactose, 6-sialyllactose, disialyllacto-N-tetraose, disialyllacto-N-hexaose, and total acidic HMOs were positively associated with infant growth over the first 6 M of life. Maternal obesity is associated with changes in HMO concentrations that are associated with infant adiposity.

## 1. Introduction

Nutrition during infancy and in early childhood can significantly impact lifelong health [1]. The gold standard for infant nutrition is human milk (HM), which is biologically tailored to support infant growth and development. As such, breastfeeding has been connected to long-term health benefits for both mother and child, including reduced risk for developing obesity in the offspring [2,3]. Although the health benefits associated with breastfeeding are multifactorial and not completely understood, HM is composed of macronutrients, abundant micronutrients, and bioactive molecules [4] that have been shown to contribute to these positive effects. However, the individual HM components associated with infant adiposity have not been fully elucidated. Understanding the early life factors that may contribute to future risk of obesity are critical to obesity prevention efforts.

Pre-gravid body mass index (BMI) has been associated with changes in the macronutrient [5] and bioactive composition of HM [5,6] and an elevated BMI can negatively impact lactation. HM oligosaccharides (HMOs) make up the third most abundant class of biomolecules in HM [7] and although mostly undigested, HMOs play an important role in infant health through prebiotic and immunomodulatory actions in the infant gut [7,8,9]. Recent evidence suggests that there is an association between pre-pregnancy BMI and changes in HMO composition. For example, a low BMI has been shown to be negatively associated with HMO concentrations [7] and some studies have shown small, but significant correlations between BMI and/or obesity and HMO concentrations [10,11,12]. However, more research is needed to fully understand the potential impact of maternal BMI and body composition on HMO concentrations.

Recently, associations between several individual HMO levels and infant growth trajectories [13], weight-for-length z scores (WLZ) [14], and anthropometrics [15] after controlling for maternal BMI have been reported, suggesting a potential role for HMOs in influencing infant growth. In particular, HMO diversity and lacto-N-neotetraose (LNnT) concentrations measured at varying times postpartum (1–9 months) have consistently shown negative associations with infant growth [13,14] and adiposity [13,15], whereas positive associations have been reported between infant growth and the concentrations of 2-fucosyllactose (2’FL) [13,14]. While these data are compelling, the majority of studies have not included women with a wide range of BMIs and therefore did not incorporate women with obesity. The inclusion of women with all classes of obesity is imperative to fully understand the impact of maternal BMI on relationships between HMO concentrations and infant growth. Furthermore, identifying associations between daily HMO intakes, rather than HMO concentrations, and infant growth would strengthen the interpretability of these observations.

The goal of this study was to explore relationships between maternal BMI and HMO compositions in term HM from healthy mothers with normal-weight (NW), overweight (OW) and obesity (OB). Additionally, the daily intake of individual HMOs was estimated and used in linear mixed models to predict infant growth and adiposity during the first 6 months of life.

## 2. Materials and Methods

### 2.1. Participants and Study Design

**Figure 1:**
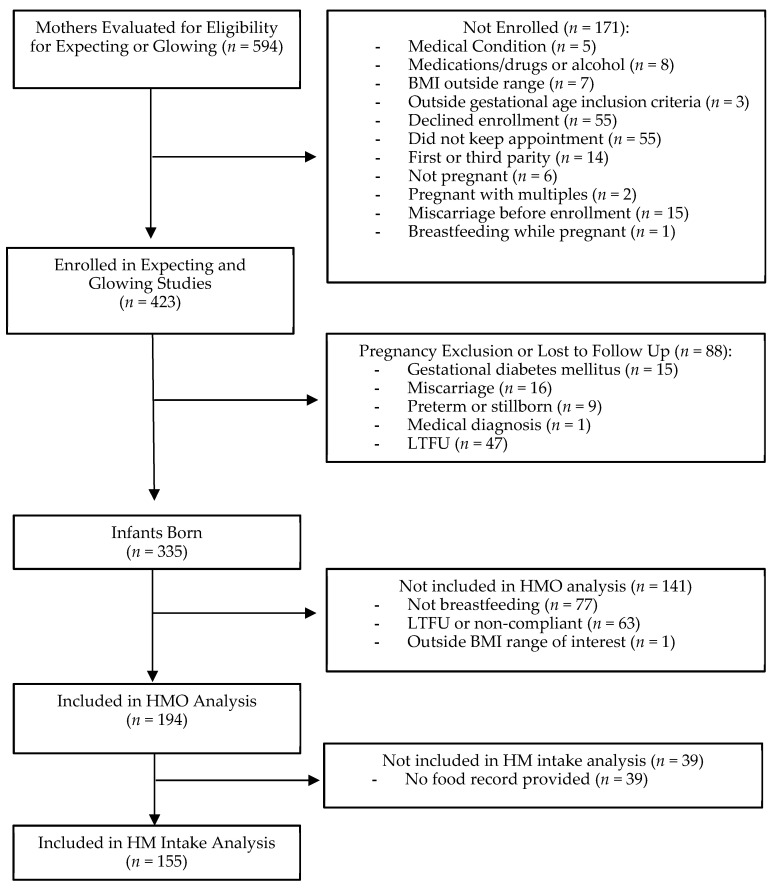
Cohort flow diagram. Breastfeeding participants (194 mother–child pairs) enrolled in two longitudinal studies (www.clinicaltrials.gov, ID# NCT01131117 (Glowing) and ID# NCT02125149 (Expecting)) were included in this study. Of the 194 participants, 39 did not provide food intake records at 2 months and were therefore not included in the intake analyses. HM = human milk, HMO = human milk oligosaccharide, and LTFU = lost to follow up.

This was a secondary analysis of breastfeeding women (194 mother–child pairs) with NW (BMI 18.32–24.96 kg/m^2^, *N* = 68), OW (BMI 25.09–29.9 kg/m^2^, *N* = 51), or OB (BMI 30.0–59.02 kg/m^2^, *N* = 75) that were enrolled in two longitudinal studies (www.clinicaltrials.gov, ID# NCT01131117 and ID# NCT02125149) (Figure 1). Enrollment criteria have been previously described in detail elsewhere for NCT01131117 [16]. Please see [5,17,18] for previously published data on human milk analyses from these cohorts. Women were recruited prior to pregnancy or during the first trimester of pregnancy. Women attended their first visit within the first 14 weeks of gestation (mean = 10 weeks with a range of 4–14 weeks). Women were excluded from the studies if they had pre-existing or ongoing medical conditions (e.g., diabetes mellitus, hypertension), used medications during pregnancy that are known to influence fetal growth, smoke, or drank alcohol. The study procedures were in accordance with the ethical standards of the Institutional Review Board of the University of Arkansas for Medical Sciences.

### 2.2. Human Milk Collection

Participants were asked to collect a human milk sample at the second feeding of the day (or before 9 am) by fully expressing one breast at postnatal age 2 months to minimize diurnal as well as hind vs. fore milk variations. An aliquot (5–25 mL) was obtained after gently mixing the sample and frozen for future analyses. The samples were stored at −70 °C and shipped to the University of California (San Diego, CA, USA) (Dr. Lars Bode) for HMO analyses. At each visit, women filled out weighed food records (for expressed HM and formula feeding) for their infants and reported their frequency of breastfeeding.

### 2.3. Human Milk Daily Intake

The daily volume (L/day) of human milk consumed by infants (*N* = 155) was measured by obtaining the infant’s weight before and after a nursing session combined with 3-day weighed food records as described above. Data from visits that lacked dietary intake data were not used in the daily intake analyses but HMO composition was still considered.

### 2.4. HMO Analysis

Concentrations of HMOs (μg/mL) were measured by high-performance liquid chromatography on an amide −80 column (2 μm particle size, 2 mm ID, 15 cm length) with fluorescent detection as previously described [19]. The absolute quantification of the following 19 HMOs was determined using the non-HMO oligosaccharide raffinose as an internal standard added to all milk samples at the beginning of analysis: 2’FL, 3-fucosyllactose (3 FL), 3-sialyllactose (3’SL), 6-sialyllactose (6’SL), difucosyllactose (DFLac), difucosyllacto-N-hexaose (DFLNH), difucosyllacto-N-tetrose (DFLNT), disialyllacto-N-hexaose (DSLNH), disialyllacto-N-tetraose (DSLNT), fucodisialyllacto-N-hexaose (FDSLNH), fucosyllacto-N-hexaose (FLNH), lacto-N-fucopentaose (LNFP) I, LNFP II, LNFP III, lacto-N-hexaose (LNH), LNnT, lacto-N-tetrose (LNT), sialyl-lacto-N-tetraose b (LSTb), and sialyl-lacto-N-tetraose c (LSTc).

### 2.5. Maternal Anthropometrics and Gestational Weight Gain

During the first study visit, maternal weight and height were measured with a standing digital scale (Tanita Corporation, Tokyo, Japan) and a wall-mounted stadiometer (Perspective Enterprises, Portage, Michigan), respectively. BMI was computed as kg/m^2^. Gestational weight gain (GWG) was calculated based upon the differences in measured weights between the participant’s first study visit and week 36 of gestation. Four of the 194 subjects did not attend their 36 weeks visit and therefore GWG for these subjects is not presented. The 2009 Institute of Medicine (IOM) guidelines for GWG based on BMI [20] adjusted for 36 weeks were used to evaluate the number of women categorized as having inadequate (less than the recommended weight gain for BMI category), adequate (within the recommended weight gain for BMI category), and excessive (exceeding the recommended weight gain for BMI category) weight gain.

### 2.6. Infant Body Composition

At postnatal research visits 2 months and 6 months, infant weight and length were measured using a tared scale (Seca, Hamburg, Germany) and a length board with a sliding foot piece (Perspective Enterprises, Portage, MI, USA), respectively. Highly trained research assistants performed these measures where the Lin’s concordance coefficient for inter-rater reliability was 0.999 during the length of the measures presented in this analysis. Infants’ gestational weight-for-age categories (small for gestational age (SGA), appropriate for gestational age (AGA), and large for gestational age (LGA)) were calculated using the updated US-based birth weight for gestational age reference [21]. WLZ and Weight-for-age (WAZ) Z-scores were calculated based on the WHO Child Growth Standards [22]. Infant fat and fat free mass (FM and FFM, respectively) were obtained using quantitative nuclear magnetic resonance (EchoMRI-AH, Echo Medical Systems, Houston, Texas) as previously described [23].

### 2.7. Maternal Plasma Insulin and Glucose

At 36 weeks of gestation and following an overnight fast, maternal blood was sampled from an antecubital vein. Plasma glucose concentrations were measured using an RX Daytona^®^ clinical analyzer (Randox Laboratories–US Limited, Kearneysville, WV, USA) and plasma insulin concentrations were measured using multispot assay kit (MesoScale Diagnostics, Rockville, MD, USA). The updated homeostasis model assessment-2 calculator from the Oxford Centre for Diabetes, Endocrinology and Metabolism was used to estimate insulin resistance (HOMA2 IR).

### 2.8. Self-Reported Outcomes

Maternal race and age were self-reported. At 0.5 months postpartum, mothers reported their infant’s sex, birth weight and their delivery mode. Gestational age was calculated using the mother’s last menstrual period and the child’s date of birth.

### 2.9. Statistical Analysis

Descriptive statistics (mean and standard error of the mean and counts and percent) were calculated for demographic data, clinical characteristics, metabolic measures and HMO concentrations. One-way ANOVAs and Pearson’s chi-squared tests were used to compare values between groups (NW, OW, and OB) for continuous and categorical data, respectively. Significance was set at alpha ≤ 0.05. Multiple comparisons tests were used to identify significant differences between the three groups. Linear models were constructed to determine (1) maternal predictors (BMI) of HMO concentrations after adjusting for maternal age, GWG, maternal secretor status, maternal fasting glucose (mg/dL) at 36 weeks of pregnancy, and maternal race as covariates; (2) associations between infant intake (g/day) of individual HMOs and infant FM or infant FFM at 2 months (kg) after controlling for infant birth weight (kg), infant sex, infant age, mode of delivery, maternal secretor status, and maternal BMI; and (3) associations between infant intake (g/day) of individual HMOs and infant WLZ or infant WAZ at 2 months (kg) after controlling for infant birth weight (kg), maternal secretor status, and maternal BMI. Linear mixed-effects models were also performed using HMO intake at 2 months to predict infant growth (FM, FFM, WLZ, and WAZ) over the first 6 months of life (time points for growth measures included 2 months and 6 months). Covariates for linear mixed-effects models predicting FM and FFM included infant birth weight (kg), infant sex, infant age (days) at time of measurement, maternal BMI (kg/m^2^), mode of delivery, and maternal secretor status, while covariates for linear mixed-effects models predicting WLZ and WAZ included infant birth weight (kg), maternal BMI (kg/m^2^), mode of delivery, and maternal secretor status. All statistical analyses other than the linear mixed-effects models were performed using IBM SPSS© Statistics version 27. Linear mixed-effects models were conducted in R (version 3.6.0) [24] using the lme4 package (version 1.1.23) [25].

## 3. Results

### 3.1. Maternal and Infant Characteristics

Maternal demographics and clinical characteristics are depicted in Table 1. As designed, maternal BMI in early pregnancy significantly differed between all three groups (*p* < 0.001). Approximately 73% of the total population was phenotypically determined to be secretors, based on a human milk 2’FL concentration of >100 nmol/mL at 2 months postpartum. A greater proportion of women with OW (31.4%) or OB (33.3%) were phenotyped as non-secretors compared to women with NW (16.2%), consistent with our previous findings [17]. The overall cohort consisted mostly of Caucasian women (81.4%) who were approximately 30 years of age. However, the proportion of non-Caucasian women who were also OW (9.8%) was significantly less than those with NW (13.2%) or OB (29.3%). The mean fasting glucose levels at 36 weeks of pregnancy progressively increased with maternal BMI category where women with OB had on average a ~10% higher value than women with NW (*p* = 0.006). There was not a significant difference in fasting insulin levels between groups, likely a result of the large variability in values. However, HOMA2 IR levels significantly increased with maternal BMI category, more than doubling in values between women with NW and those with OB (*p* < 0.001), suggesting that women with OW and OB had decreased insulin sensitivity. Over the course of pregnancy, women with OB gained significantly less weight on average compared to women with NW or OW (*p* < 0.001), despite the fact that ~37% of women with NW had “inadequate” weight gain and greater proportions of women with OW and OB (62.7% and 49.3% vs. 10.3%, respectively, *p* < 0.001) gained an “excessive” amount of weight according to the IOM GWG recommendations [20]. The mean gestational age was 39 weeks for all groups and women with OW or OB tended to have slightly more C-sections than did women with NW (*p* = 0.091).

Infant characteristics are depicted in Table 2. The proportions of male and female infants were similar across groups. Birth weights did not differ between groups and approximately 80% of the infants from each group were categorized as AGA. Infant growth parameters measured at 2 and 6 months (Table 2) indicate no differences in infant growth at these time points (Table 2). Of the infants whose HM intakes were recorded (*N* = 155), 15 infants were supplemented with formula while 140 were exclusively breastfed. Infants from all three groups consumed similar mean amounts of total milk (~662 mL), HM (~642 mL), and formula milk (~195 mL) at 2 months postpartum.

### 3.2. Maternal BMI Is Associated with HMO Concentrations

Several HMO concentrations differed in milk from NW, OW, and OB groups within each of the non-secretor and secretor phenotypes (Table 3). Many of the observed differences were also dependent on secretor status. In non-secretors, 3 sialylated HMO concentrations differed significantly between groups (3’SL, DSLNH, FDSLNH), where concentrations of 3’SL were significantly lower in women with OW compared to those with NW (28% lower) or OB (39.4% lower) and ~30–45% lower concentrations of DSLNH and FDSLNH were observed in women with OB, compared to other groups (Table 3). Together, these data suggest that HMO sialylation may be negatively associated with maternal adiposity in women who are non-secretors.

In women phenotyped as secretors, over 50% of the measured HMO concentrations were significantly different across maternal BMI groups (Table 3). All three of the neutral HMO concentrations differed between groups. Women with OW had ~22% lower concentrations of HM LNT and 27% and 53% lower concentrations of HM LNnT compared to women with NW and OB, respectively, whereas women with OB had elevated concentrations of HM LNnT (~53% higher) but ~20–30% lower concentrations of HM LNH compared to women with NW and OW. Interestingly, when the total concentrations of neutral HMOs were considered, women with OB had significantly higher concentrations in their milk compared to the other groups, albeit these differences were quite small (3–4% higher, *p* = 0.014). Concentrations of several fucosylated HMOs were also altered between maternal BMI groups. Namely, women with OB had higher levels of 3 FL (~35–40% higher) but lower concentrations of DFLNT (~20%), FLNH (~30%) and DFLNH (~48%) in HM compared to secretor women with NW. Women with OW had HMO concentrations that fell in the middle of these two groups (Table 3). Several sialylated HMO concentrations were lower in secretor women with OB, compared to those with NW or OW. The concentrations of DSLNT, DSLNH, and FDSLNH were ~27%, ~45–50%, and ~37% lower in HM from women with OB, compared to those with NW or OW (Table 3), supporting the notion that maternal adiposity may have a negative association with HMO sialylation, regardless of secretor status. Accordingly, when the total concentrations of measured acidic HMOs were considered, secretor women with OB tended to have ~10% lower levels (*p* = 0.075) in their HM than those with NW or OW. A similar decrease (~12%) was also observed in non-secretor women with OB. However, significance was not obtained likely as a result of the smaller sample sizes for the non-secretors.

To determine the relationship between maternal BMI and HMO concentrations, linear models were developed to adjust for maternal secretor phenotype, maternal age, maternal race, and third trimester fasting glucose levels (Table 4). Maternal BMI was a significant positive predictor for LNnT (β = 3.9, *p* < 0.001), 3 FL (β = 30.6, *p* = 0.016), and 6’SL (β = 6.8, *p* = 0.042) but a negative predictor of DFLNT (β = −23.9, *p* = 0.001), DSLNT (β = −11.9, *p* < 0.001), DSLNH (β = −5.4, *p* < 0.001), and FDSLNH (β = −8.7, *p* = 0.006) levels at 2 months postpartum. Increasing maternal BMI was also significantly associated with lower total amounts of acidic HMOs (β = −12.6, *p* = 0.044) supporting the hypothesis that maternal adiposity may negatively impact HMO sialylation.

### 3.3. HMO Intake Differs from Infants Born to Women with NW vs. OW vs. OB

**Figure 2:**
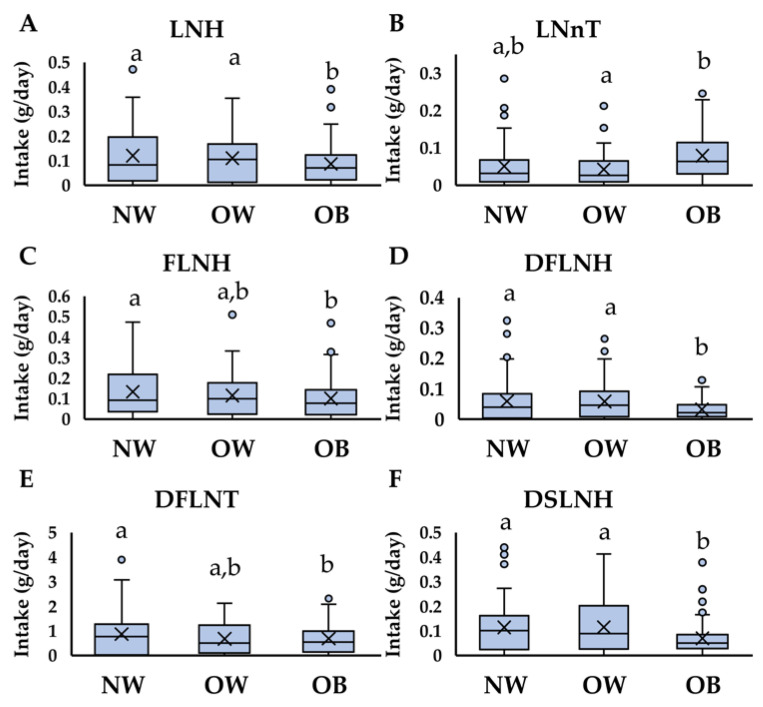
HMO intake at 2 months of age differs between infants born to women with NW, OW, and OB. HMO intakes (mg/day) were estimated at 2 months of age from infants (*N* = 155) born to women with normal-weight (NW) overweight (OW) or obesity (OB). One-way ANOVAs were performed to identify HMO intakes that differed significantly between groups, where *p* < 0.05. Differing letters indicate groups with significantly different mean values. (**A**) Lacto-N-hexaose (LNH), (**B**) lacto-N-neotetraose (LNnT), (**C**) fucosyllacto-N-hexaose (FLNH), (**D**) difucosyllacto-N-hexaose (DFLNH), (**E**) difucosyllacto-N-tetrose (DFLNT), and (**F**) disialyllacto-N-hexaose (DSLNH). Box plots indicate the median (central line), the mean (the x in the center of the box), the interquartile range (distance from top of box to bottom of box), the minimum and maximum values (top and bottom of the bar, respectively) and any outliers (dots that fall outside of the min and max).

As noted above, the daily intake of human milk did not differ between infants born to women in each BMI category (Table 2), and therefore differences in individual infant intakes of HMOs between groups (Figure 2) were consistent with the observed differences in HMO concentrations (Table 3). Infants born to women with OB consumed lesser amounts of LNH (*p* = 0.007), FLNH (*p* = 0.02), DFLNH (*p* < 0.001), DFLNT (*p* = 0.02), and DSLNH (*p* < 0.001) compared to both women with NW and OW (for LNH, DFLNH, and DSLNH) or to women with NW (for FLNH and DFLNT). LNnT consumption was the greatest in infants born to women with OB, compared to the other groups (*p* = 0.043, Figure 2). Interestingly, the total infant intakes of acidic HMOs did not differ across groups as would be expected based on the findings that total acidic HMO concentrations decreased as BMI increased (Table 4).

### 3.4. Infant HMO Intake Is Associated with Infant Body Composition and Weight

Linear mixed-effects models were then conducted to determine whether HMO intakes were predictors of infant growth over the first 6 months of life (Table 5). While no significant associations between HMO intakes and FFM occurred between 2 and 6 months, 3 FL, LNFPII, LSTb, and DSLNH showed positive associations with infant FM, WLZ, and WAZ (Table 5). 3’SL intake was positively associated with FM and WAZ and LNFP III, 6’SL and DSLNT were positively associated with FM only. Finally, the intakes of total acidic HMOs and total HMOs were positively associated with infant growth between 2 and 6 months (FM, WLZ, and WAZ, Table 5).

To determine whether formula feeding contributed to these observed relationships, linear mixed-effects models were conducted in exclusively breastfed infants only (*N* = 140). In exclusively breastfed infants, all associations between HMO intakes and infant FM remained significant, other than for DSLNT where the p value increased to a trend (*p* = 0.057). Only LNFP II and DSLNH intakes maintained significant associations with WFZ in exclusively breastfed infants, whereas all associations between HMO intakes and WAZ remained significant other than LNFP III (*p* = 0.093).

## 4. Discussion

In the largest cohort to date of women whose BMIs span a range that includes those with normal weight, overweight, and all classes of obesity, this study aimed to scrutinize the effect of maternal BMI on HMO concentrations and infant HMO intakes at 2 months postpartum. To our knowledge, we are the first to report daily infant HMO intakes, as a more specific measure of HMO exposure, to explore the relationships between HMOs and infant growth over the first 6 months of life. The data presented herein indicate that maternal BMI was positively associated with LNnT, 3 FL, and 6’SL concentrations at 2 months postpartum. While LNnT intake did not predict infant growth outcomes, both 3 FL and 6’SL were positively associated with infant FM over the first 6 months of life, suggesting that these HMOs may be promising targets for future work in human milk nutritional programing. On the other hand, several sialylated HMO concentrations (DSLNT, DSLNH, and FDSLNH) and the total levels of acidic HMOs at 2 months postpartum were negatively associated with maternal BMI, while some of the same HMOs (DSLNT, DSLNH and total acidic HMOs) were positively associated with infant growth parameters. As no significant differences were observed in infant body composition, WLZ, or WAZ at 2–6 months postpartum between maternal BMI groups one might hypothesize that decreasing concentrations of sialylated/acidic HMOs with increasing maternal BMI may provide protective mechanisms via human milk intake against excess weight gain and adiposity in offspring. Together, these data provide solidifying evidence that contributes to the existing body of knowledge showing a relationship between maternal BMI and HMO composition, while expanding the field with reports of infant HMO intakes and data describing the relationship between infant HMO exposure and growth.

Maternal genetics (expression patterns of Secretor (Se) and Lewis (Le) gene alleles, which code for different fucosyltransferases) have the greatest impact on HMO composition [26]. However, we and several others have concluded that various maternal characteristics (age, race, ethnicity, and parity) including pre-pregnancy BMI [10,11,17] and maternal glucose homeostasis [17] can affect HMO composition during lactation. To our knowledge, this study presented herein is one of the first to explore the relationship between maternal BMI and HMO composition in a large group of women whose BMIs encompassed Class I–Class III obesity. In a recent publication, Langström et al. reported a significant association between maternal BMI and HMO concentrations at 3 months postpartum in one of the largest cohorts to date (STEPS Study, [14]). In contrast to our observations, Langström et al. identified a negative relationship between maternal BMI and LNnT after adjusting for maternal and infant characteristics. However, the participants from the STEPS Study did not have obesity (median BMI = 23.0, [14]), which may explain the observed discrepancy between these two studies. Consistent with our observations, a positive association between pre-pregnancy BMI and LNnT concentrations at 2–8 days and at 88–119 days postpartum was recently reported in a Brazilian cohort that included women with overweight and obesity [11], suggesting that the relationship between BMI and LNnT concentrations might change when higher BMIs are included in the analysis. LNnT consumption can promote positive health benefits to the infant gut [27] and therefore identifying maternal predictors of LNnT concentrations may prove useful in future attempts to develop interventions for optimizing HM composition and infant nutrition.

Maternal obesity is often complicated by a myriad of metabolic changes that may contribute to altered mammary gland development [28,29] and/or variations in the production of human milk components [5]. Although the mechanisms driving the observed differences in HMO composition with increasing maternal BMI have not been elucidated, it is likely both physiological and environmental factors that contribute to altered HMO synthesis [10]. We have previously demonstrated that glucose homeostasis during pregnancy is associated with HMO composition at 2 months postpartum [17]. In particular, maternal insulin levels and measures of insulin sensitivity were associated with total HMO and sialylated HMO concentrations in non-secretors. Knowing that insulin is critical in stimulating gene expression regulating mammary differentiation and milk lactose synthesis [30], we hypothesized that dysregulation of insulin levels and/or insulin signaling at the level of mammary epithelium likely contributes to HMO production and therefore concentrations. Interestingly, total human milk lactose concentration does not seem to be associated with maternal %FM [31], and may therefore be more specific to maternal insulin signaling rather than maternal adiposity. Alternatively, lifestyle changes such as changes in the maternal diet may also contribute to the observed differences in HMO concentrations across maternal BMIs. Azad et al. recently evaluated the relationship between a healthy eating index (HEI) score [32] and HMO compositions in milk from a large, mostly Caucasian, healthy cohort of lactating women [10]. Although they reported no associations between HEI and total HMO content, they found weak, but significant associations between several HMOs (FLNH, LSTb, LNT, and DFLNH) and individual dietary components [10].

While the health benefits most commonly associated with HMOs relate to preventing infectious disease [7], recent evidence also suggests an association between HMO concentrations and infant growth. Notably, a decreased concentration of sialylated HMOs has been found in HM from mothers with growth-stunted infants [33] suggesting a potential growth-supportive role for sialylated HMOs. However, this relationship might not always be helpful to the infant. Our data suggest that sialylated/acidic HMOs are positively associated with infant growth in a healthy population of infants. In particular, 6’SL was identified as one of the 2 HMO intakes that predicted increased infant FM and was also associated with elevations in maternal BMI, pinpointing 6’SL as a probable human milk candidate linking maternal obesity to infant fat accretion in the postnatal period. 3 FL was also associated with infant growth and maternal BMI, suggesting that it may also be involved in maternal obesity-related postnatal nutritional programming. In a pilot study, Larsson et al. explored the association between HMO concentrations and excessive infant weight gain and found positive associations between total HMO concentrations as well as total HMO-bound fucose and both infant FM index and weight velocity over the first 5 months postpartum [13]. Specifically, a positive association between 2’FL concentrations in HM from secretors and infant growth has been reported [13,14], suggesting that increased fucosylated HMOs might promote excess weight gain and adiposity early in life. In a similarly sized study, Alderete et al. reported a positive association between several HMOs (DSLNT, LNFP II and FDSLNH) and infant FM at 6 months of age [15]. Between 2 and 6 months postpartum, we also found positive associations between infant intakes of fucosylated HMOs (LNFP II, LNPF III, and 3 FL) and infant body composition and growth, supporting the proposed role for fucosylated HMOs in promoting early life adiposity. Interestingly, there were no significant associations between LNnT or 2’FL and infant growth parameters in the current study, differing significantly from those previously published [13,14,15]. It is likely that the uniqueness of the present study, such as the inclusion of both secretors and non-secretors in all analyses, the addition of women with a large range in BMI, and the evaluation of infant HMO intakes, has led to these discrepancies.

There is little available data describing the potential mechanisms for infant growth promotion via HMOs, as this field is still very new. However, the data that do exist strongly suggest a mediatory role for the infant gut microbiome, as HMOs are not digested by the human gut but are critical to the establishment a healthy infant microbiome [34]. One report in particular did an exemplary job demonstrating a causal microbiota-dependent relationship between sialylated oligosaccharides and offspring growth via a greater ability to utilize nutrients for anabolism [33]. Charbonneau et al. discovered that breastfeeding Malawian women whose infants showed stunted growth had significantly less concentrations of fucosylated, sialylated, and total HMOs than human milk from women whose infants were thriving [33]. Using both mouse and piglet gnotobiotic models colonized with or without the feces from a growth-stunted infant they tested the effects of a Malawian diet supplemented with or without sialylated bovine milk oligosaccharides to promote growth. They discovered that offspring supplemented with sialylated oligosaccharides had improved growth that was characterized by augmented lean mass gain, altered bone development, and metabolic changes that were indicative of normal substrate utilization patterns [33]. Importantly, these improvements were microbiota-dependent, as the animals that were not colonized did not see the same effects [33]. Although these data strongly suggest a growth-promoting role for sialylated HMOs in growth-stunted infants, the mechanisms for the observed associations between HMOs and body composition or growth in healthy infants have not been established. One study suggests that HMO composition may influence infant feeding behavior, which could directly influence growth via changes in caloric intake [35]. Additionally, previous studies have observed HMOs and HMO metabolites in the urine of breastfed infants, suggesting that some HMOs may have systemic effects on infant physiology [36,37]. Future studies that aim to dissect the mechanisms linking HMOs to infant growth should consider these possibilities in addition to the influence of the infant microbiome.

Although the data presented herein and those presented previously [13,14,15] provide a compelling argument for the influence of HMOs on infant growth, human milk components are consumed as a complete matrix and not as individual parts. Therefore, studies that consider the impact of maternal BMI and/or obesity on human milk composition as a full matrix may offer further insight into how human milk participates in nutritional programming. For example, we and others [5,6,38,39] have shown that maternal obesity is associated with changes in human milk macronutrients (e.g., lipids and proteins) and obesity-related bioactive components (insulin [38], leptin [6], and c-reactive protein [39]) over the course of lactation that are related to infant growth and body composition. It is probable that these changes work in concert with differing HMO concentrations to provide the overall effect on postnatal nutritional programing. ‘Omics’ studies (metabolomics, proteomics, lipidomics, etc.) performed on human milk samples have provided a more comprehensive approach to understanding the impact of maternal BMI on milk composition [18,40,41,42]. However, very few have combined multiple omics techniques to gain understanding of the human milk matrix as a whole. Furthermore, longitudinal studies following infant outcomes that are matched to human milk omics analyses are severely lacking. Future works using this type of comprehensive approach will be necessary to define the role of human milk in nutritional programing.

There are several limitations to this study that should be considered. First, the data presented here are observational and must be interpreted within the context of this limitation. However, our ability to estimate daily HM intake has provided a mechanism to better estimate the association between HMO exposures and infant growth than what could be found with using HMO concentrations alone. Secondly, while this study excels in the inclusion of women with a wide range of BMIs, it lacks in racial diversity and therefore the generalizability may be limited to populations with a racial makeup similar to Arkansas. This study is, however, one of few that have evaluated a large enough population to allow for the consideration of maternal secretor status throughout the analysis, adding to the external validity. Finally, this study is limited to the assessment of infant growth through 6 months, which may not be directly reflective of obesity risk as the children get older. Future longitudinal studies should aim to evaluate the relationship between HMO consumption and risk of obesity later in life.

In conclusion, the findings presented herein help to solidify the relationship between maternal BMI and HMO concentrations in mature HM and suggest that infants born to women with OB consume differing amounts of HMOs. Furthermore, these data indicate a positive association between HMO consumption and both infant adiposity and growth that exists when maternal BMI and secretor phenotype are controlled for. This observational study sets a precedence for future work that aims to evaluate a direct link between HMO consumption and infant growth within the context of maternal obesity.

## Figures and Tables

**Table 1:** Maternal Characteristics.

	NW	OW	OB	Total	*p* Value
	*N* = 68	*N* = 51	*N* = 75	*N* = 194	
* BMI (kg/m^2^)					<0.001 ^1^
Mean (SE)	22.2 ± 0.2 ^a^	27.4 ± 0.2 ^b^	36.2 ± 0.6 ^c^	28.9 ± 0.5	
* Secretor Status (*N* (%))					0.05 ^2^
Non-secretor	11 (16.2%) ^a^	16 (31.4%) ^a^	25 (33.3%) ^a^	52 (26.8%)	
Secretor	57 (83.8%) ^a^	35 (68.6%) ^a^	50 (66.7%) ^a^	142 (73.2%)	
* Race (*N* (%))					0.008 ^2^
Non-Caucasian	9 (13.2%) ^a,b^	5 (9.8%) ^a^	22 (29.3%) ^b^	36 (18.6%)	
Caucasian	59 (86.8%) ^a,b^	46 (90.2%) ^a^	53 (70.7%) ^b^	158 (81.4%)	
Age (years)					0.99 ^1^
Mean (SE)	30.6 ± 0.4	30.5 ± 0.4	30.5 ± 0.5	30.5 ± 0.3	
* Fasting Glucose (mg/dL)					0.006 ^1^
Mean (SE)	79.1 ± 2.2 ^a^	84.2 ± 2.2 ^a,b^	88.4 ± 1.7 ^b^	84.8 ± 1.2	
Fasting Insulin (pmol/L)					0.32 ^1^
Mean (SE)	68.8 ± 29.1	99.8 ± 24.7	109.2 ± 7.3	96.3 ± 10.8	
* HOMA2 IR					<0.001 ^1^
Mean (SE)	0.7 ± 0.1 ^a^	1.0 ± 0.1 ^b^	1.9 ± 0.1 ^c^	1.4 ± 0.1	
* Gestational Weight Gain (kg)					<0.001 ^1^
Mean (SE)	12.4 ± 0.3 ^a^	12.6 ± 0.6 ^a^	9.2 ± 0.5 ^b^	11.2 ± 0.3	
IOM GWG * Recommendation (*N* (%))					<0.001 ^2^
Inadequate	25 (36.8%) ^a^	4 (7.8%) ^b^	12 (16.0%) ^b^	41 (21.1%)	
Adequate	35 (51.5%) ^a^	15 (29.4%) ^b^	23 (30.7%) ^b^	73 (37.6%)	
Excessive	7 (10.3%) ^a^	32 (62.7%) ^b^	37 (49.3%) ^b^	76 (39.2%)	
Unknown	1 (1.5%) ^a^	0 ^a^	3 (4.0%) ^a^	4 (2.1%)	
Gestational Age (weeks)					0.19 ^1^
Mean (SE)	39.2 ± 0.1	39.5 ± 0.1	39.1 ± 0.2	39.2 ± 0.1	
Delivery Mode					0.09 ^2^
C-section	18 (26.5%)	18 (35.3%)	33 (44.0%)	69 (35.6%)	
Vaginal	50 (73.5%)	33 (64.7%)	42 (56.0%)	125 (64.4%)	

^1^ One-way ANOVA. ^2^ Pearson′s chi-squared test. * Each differing superscript letter denotes values that are significantly different between groups. NW = normal weight, OW = overweight, and OB = obese.

**Table 2:** Infant Characteristics.

	NW	OW	OB	Total	*p* Value
	*N* = 68	*N* = 51	*N* = 75	*N* = 194	
Infant Sex (*N* (%))					0.46 ^2^
Female	29 (42.6%)	22 (43.1%)	39 (52.0%)	90 (46.4%)	
Male	39 (57.4%)	29 (56.9%)	36 (48.0%)	104 (53.6%)	
Birth Weight (kg)					0.29 ^1^
Mean (SE)	3.5 ± 0.1	3.6 ± 0.1	3.4 ± 0.1	3.5 ± 0.0	
Size for Gestational Age (*N* (%))					0.57 ^2^
SGA	3 (4.4%)	0	2 (2.7%)	5 (2.6%)	
AGA	56 (82.4%)	41 (80.4%)	61 (81.3%)	158 (81.4%)	
LGA	9 (13.2%)	10 (19.6%)	12 (16.0%)	31 (16.0%)	
Height (cm)					
2 M, Mean (SE)	56.9 ± 0.3	57.1 ± 0.3	56.7 ± 0.3	56.9 ± 0.2	0.53 ^1^
6 M, Mean (SE)	65.8 ± 0.3	65.8 ± 0.3	65.1 ± 0.3	65.5 ± 0.2	0.20 ^1^
Weight (kg)					
2 M, Mean (SE)	5.3 ± 0.1	5.4 ± 0.1	5.1 ± 0.1	5.3 ± 0.1	0.14 ^1^
6 M, Mean (SE)	7.6 ± 0.1	7.6 ± 0.1	7.5 ± 0.11	7.6 ± 0.1	0.63 ^1^
Fat Mass (kg)					
2 M, Mean (SE)	1.1 ± 0.0	1.2 ± 0.1	1.1 ± 0.0	1.1 ± 0.0	0.34 ^1^
6 M, Mean (SE)	2.2 ± 0.1	2.3 ± 0.1	2.3 ± 0.1	2.2 ± 0.1	0.33 ^1^
Fat Mass (%)					
2 M, Mean (SE)	20.0 ± 0.5	20.9 ± 0.6	20.8 ± 0.4	20.6 ± 0.3	0.28 ^1^
6 M, Mean (SE)	28.3 ± 0.6	30.0 ± 0.9	29.1 ± 0.8	29.1 ± 0.4	0.32 ^1^
Lean Mass (kg)					
2 M, Mean (SE)	3.5 ± 0.1	3.6 ± 0.1	3.55 ± 0.1	3.6 ± 0.0	0.61 ^1^
6 M, Mean (SE)	4.6 ± 0.1	4.7 ± 0.1	4.73 ± 0.1	4.7 ± 0.0	0.49 ^1^
Infant Intake	*N* = 55	*N* = 43	*N* = 57	*N* = 155	
Human Milk + Formula (mL), Mean (SE)	675.8 ± 47.0	639.4 ± 45.2	664.4 ± 43.7	661.5 ± 26.2	0.86 ^1^
Human Milk (mL), Mean (SE)	667.2 ± 48.3	624.9 ± 47.5	632.5 ± 44.6	642.7 ± 27.0	0.79 ^1^
Formula Milk (mL), Mean (SE)	158.4 ± 28.5	207.9 ± 82.3	202.2 ± 38.1	194.6 ± 27.2	0.82 ^1^

^1^ One-way ANOVA. ^2^ Pearson′s chi-squared test. SGA = small for gestational age, AGA = appropriate for gestational age, LGA = large for gestational age, NW = normal weight, OW = overweight, and OB = obese.

**Table 3:** Human Milk Oligosaccharide (HMO) Concentrations (μg/mL) at 2 Months Postpartum.

	Non-Secretor				Secretor			
HMO, μg/mL	NW (*n* = 11) Mean ± SE	OW (*n* = 16) Mean ± SE	OB (*n* = 25) Mean ± SE	*p* Value ^1^	NW (*n* = 57) Mean ± SE	OW (*n* = 35) Mean ± SE	OB (*n* = 50) Mean ± SE	*p* Value ^1^
* LNT	902.7 ± 104.6	1026.6 ± 121.4	1215.2 ± 171.6	0.41	1095.5 ± 51.5 ^a^	870.0 ± 66.5 ^b^	1153.0 ± 72.5 ^a^	0.01
* LNnT	80.0 ± 10.6	70.4 ± 12.2	118.1 ± 15.7	0.05	100.5 ± 7.9 ^a^	73.3 ± 5.9 ^b^	154.7 ± 11.5 ^c^	<0.001
* LNH	284.3 ± 77.3	162.1 ± 26.3	159.6 ± 40.1	0.17	204.1 ± 13.2 ^a,b^	237.8 ± 30.0 ^a^	161.0 ± 15.7 ^b^	0.02
2’FL	6.7 ± 1.6	2.4 ± 0.6	12.8 ± 6.4	0.36	885.2 ± 61.4	1129.8 ± 104.3	884.5 ± 75.9	0.06
* 3 FL	2140.8 ± 169.9	2218.9 ± 148.8	2447.0 ± 205.3	0.52	1595.4 ± 92.6 ^a^	1669.4 ± 123.7 ^a^	2249.3 ± 187.0 ^b^	0.002
DFLac	10.1 ± 8.4	19.0 ± 7.3	25.5 ± 13.2	0.70	247.8 ± 14.5	286.3 ± 23.4	229.1 ± 12.7	0.07
LNFP I	109.3 ± 8.4	121.5 ± 8.9	117.0 ± 8.9	0.72	1260.7 ± 99.3	1207.7 ± 134.9	1208.7 ± 140.5	0.94
LNFP II	1842.5 ± 152.9	2073.2 ± 92.0	1919.4 ± 127.0	0.51	1416.0 ± 53.0	1358.5 ± 64.2	1536.1 ± 74.7	0.17
LNFP III	34.5 ± 6.3	29.9 ± 5.4	32.4 ± 7.5	0.93	47.4 ± 3.1	56.4 ± 10.0	40.2 ± 3.0	0.11
* DFLNT	428.9 ± 58.9	511.5 ± 62.4	364.6 ± 44.2	0.14	1900.6 ± 96.5 ^a^	1723.5 ± 109.2 ^a,b^	1524.9 ± 98.9 ^b^	0.02
* FLNH	297.9 ± 67.4	171.9 ± 29.6	175.2 ± 31.5	0.09	238.8 ± 13.2 ^a^	215.9 ± 19.6 ^a,b^	167.3 ± 15.1 ^b^	0.003
* DFLNH	48.3 ± 8.1	40.1 ± 4.5	32.8 ± 5.6	0.23	159.2 ± 16.7 ^a^	155.1 ± 20.2 ^a^	81.0 ± 12.4 ^b^	0.001
* 3’SL	162.8 ± 12.9 ^a^	117.2 ± 12.3 ^b^	193.5 ± 14.8 ^a^	0.001	343.3 ± 18.1	387.7 ± 28.8	409.4 ± 32.4	0.16
6’SL	369.5 ± 41.4	397.2 ± 45.0	503.0 ± 56.7	0.19	555.5 ± 26.9	594.4 ± 53.6	649.7 ± 43.4	0.21
LSTb	80.4 ± 5.2	120.9 ± 20.1	113.4 ± 11.9	0.22	112.6 ± 6.4	112.0 ± 6.7	119.2 ± 9.8	0.78
LSTc	54.2 ± 7.3	51.3 ± 8.0	94.3 ± 16.0	0.05	185.1 ± 11.3	179.2 ± 14.2	191.8 ± 17.2	0.84
* DSLNT	335.8 ± 41.7	333.7 ± 28.9	280.3 ± 24.9	0.30	518.5 ± 30.2 ^a^	471.9 ± 36.7 ^a,b^	377.2 ± 36.9 ^b^	0.01
* DSLNH	174.4 ± 28.9 ^a^	145.9 ± 16.9 ^a^	96.0 ± 14.4 ^b^	0.01	230.0 ± 14.3 ^a^	256.6 ± 21.8 ^a^	127.4 ± 14.0 ^b^	<0.001
* FDSLNH	837.5 ± 85.0 ^a^	664.8 ± 83.0 ^ab^	483.4 ± 70.2 ^b^	0.02	433.7 ± 30.7 ^a^	431.7 ± 35.6 ^a^	270.9 ± 28.2 ^b^	<0.001
* Neutral HMO	6185.8 ± 163.2	6447.5 ± 105.3	6619.7 ± 188.8	0.28	9151.3 ± 65.2 ^a^	9039.7 ± 103.2 ^a^	9389.8 ± 86.4 ^b^	0.01
Acidic HMO	2014.6 ± 135.0	1830.9 ± 80.6	1763.8 ± 92.3	0.27	2378.7 ± 48.5	2471.0 ± 92.1	2228.1 ± 82.3	0.08
Total HMO	8200.4 ± 81.7	8278.4 ± 51.8	8383.6 ± 164.4	0.68	11,529.9 ± 56.6	11,510.7 ± 57.0	11617.9 ± 74.5	0.47

^1^ One-way ANOVA. * Each differing superscript letter denotes values that are significantly different between groups. NW = normal weight, OW = overweight, and OB = obese.

**Table 4:** Maternal BMI as a Predictor of HMO Concentrations at 2 Months Postpartum *.

HMO, μg/mL	β	*p* Value
LNT	8.5	0.20
LNnT	3.9	<0.001
LNH	−1.3	0.53
2’FL	−4.1	0.48
3 FL	30.7	0.02
DFLac	−1.7	0.18
LNFP I	0.6	0.94
LNFP II	0.9	0.87
LNFP III	<0.01	1.0
DFLNT	−23.9	0.001
FLNH	−0.8	0.61
DFLNH	−1.5	0.35
3’SL	1.6	0.46
6’SL	6.8	0.04
LSTb	−0.3	0.72
LSTc	1.1	0.30
DSLNT	−11.9	<0.001
DSLNH	−5.4	<0.001
FDSLNH	−8.69	0.006
Neutral HMO	11.3	0.18
Acidic HMO	−12.6	0.04
Total HMO	−1.3	0.84

* Linear regressions (*n* = 194 subjects) included HMO concentration (μg/mL) as the dependent variables and maternal BMI (kg/m^2^) as the predictor. Model covariates included maternal race, maternal age, maternal secretor phenotype, and maternal fasting glucose (mg/dL) at 36 weeks of pregnancy.

**Table 5:** HMO Intake Is Associated with Infant Growth at 2–6 Months of Age.

HMO Intake (g/day)	β	Lower C.I.	Upper C.I.	* *p* Value
** Infant FM, 2–6 months				
3 FL	0.07	0.02	0.12	0.006
LNFP II	0.09	0.02	0.16	0.008
LNFP III	2.11	0.12	4.11	0.04
3’SL	0.35	0.01	0.70	0.05
6’SL	0.20	0.01	0.40	0.04
LSTb	1.13	0.24	2.02	0.01
DSLNT	0.25	0.01	0.49	0.04
DSLNH	0.59	0.10	1.07	0.02
Acidic HMO	0.08	0.03	0.14	0.004
Total HMO	0.01	0.00	0.03	0.03
*** Infant WLZ, 2–6 months				
LNT	0.23	0.02	0.45	0.04
3 FL	0.17	0.03	0.30	0.02
LNFP II	0.26	0.07	0.44	0.007
LSTb	3.02	0.65	5.39	0.01
DSLNH	1.55	0.26	2.83	0.02
Acidic HMO	0.18	0.03	0.33	0.02
Total HMO	0.03	0.00	0.06	0.04
*** Infant WAZ, 2–6 months				
3 FL	0.18	0.06	0.29	0.002
LNFP II	0.21	0.06	0.37	0.008
3’SL	0.82	0.04	1.60	0.04
LSTb	2.91	0.89	4.93	0.005
DSLNH	1.17	0.06	2.27	0.04
Acidic HMO	0.18	0.05	0.30	0.006
Total HMO	0.03	0.00	0.06	0.03

* Linear mixed-effects models (*n* = 155 subjects) were performed using HMO intake at 2 months (g/day) to predict infant fat mass (FM), weight-for-length z-score (WLZ), and weight-for-age z-score (WAZ) over the first 6 months of life (time points 2 months and 6 months). ** Model covariates for FM included infant birth weight (kg), infant sex, infant age (days) at time of measurement, maternal BMI (kg/m^2^), mode of delivery, and maternal secretor status. *** Model covariates for WLZ and WAZ included infant birth weight (kg), maternal BMI (kg/m^2^), mode of delivery, and maternal secretor status.
